# Diminished sensitivity of audiovisual temporal order in autism spectrum disorder

**DOI:** 10.3389/fnint.2013.00008

**Published:** 2013-02-27

**Authors:** Liselotte de Boer-Schellekens, Mart Eussen, Jean Vroomen

**Affiliations:** ^1^Department of Cognitive Neuropsychology, Tilburg UniversityTilburg, Netherlands; ^2^Yulius Mental Health OrganizationDordrecht, Netherlands

**Keywords:** autism spectrum disorder, multisensory integration, audiovisual processing, temporal intersensory sensitivity, social (un)impairment

## Abstract

We examined sensitivity of audiovisual temporal order in adolescents with autism spectrum disorder (ASD) using an audiovisual temporal order judgment (TOJ) task. In order to assess domain-specific impairments, the stimuli varied in social complexity from simple flash/beeps to videos of a handclap or a speaking face. Compared to typically-developing controls, individuals with ASD were generally less sensitive in judgments of audiovisual temporal order (larger just noticeable differences, JNDs), but there was no specific impairment with social stimuli. This suggests that people with ASD suffer from a more general impairment in audiovisual temporal processing.

## Introduction

In the multisensory world we live in, we are constantly bombarded with information that reaches us through our different senses. The brain has to synthesize this mix of sensory information into one coherent multisensory percept. An example of this intersensory process is a speaker that can be seen and heard at the same time. This results in an ensemble of multiple features across the different senses that our brain has access to (i.e., lip movement, facial expression, speed, and temporal structure of the speech sound) that ultimately leads to an increase in perceptual reliability. This sensory synthesis is a constantly occurring phenomenon that shapes our view of the world and it is therefore crucial for our everyday, social and adaptive behavior (Wallace, 2004). It also raises the question how our brain integrates this wealth of sensory information and how a coherent representation of the world is obtained (Keetels and Vroomen, 2012). Another question is what happens if the brain is impaired in integrating this mix of sensory input.

This is one of the issues raised in contemporary research in autism spectrum disorder (ASD). In addition to traditional impairments in communication, social behavior, and stereotyped repetitive movements, abnormalities in sensory processing are often reported in ASD. Indeed, several contemporary theories on ASD reflect the idea that sensory deficits are core symptoms of autism as well (Kern et al., 2007; Crane et al., 2009). In this view, sensory deficits might have downstream effects on the development of the perceptual system that may eventually lead to adverse consequences for communication and social interaction (Mottron and Burack, 2001; Bertone et al., 2005).

Early clinical observations dating back to Kanner (1943) already emphasized sensory avoidance and a tendency to over-focus on local attributes. More recent accounts have proposed that autism is characterized by weak “central coherence” (Frith, 1989). Central coherence is the everyday tendency to process incoming information in its context, pulling information together for higher-level meaning, often at the expense of memory for detail. Frith (1989) proposed that people with autism show detail-focused local processing in which features are perceived and retained at the expense of global configuration and contextualized meaning. Some have linked this to functional and/or neuroanatomical under-connectivity between brain areas (Brock et al., 2002; Courchesne and Pierce, 2005). The distribution of attention to global/local features may be different in autism, leading one to predict relatively good performance where attention to local information is advantageous, but poor performance on tasks requiring the recognition of global meaning or integration of stimuli in context (Happé, 1999). Iarocci and McDonald (2006) suggest that many of the leading theories of autism allude to dynamic constructs and conceptualizations such as central coherence, temporal binding, shifting attention, enhanced perception, and neural modulation and connectivity that may all eventually involve multisensory processing and integration.

Empirical evidence of multisensory processing deficits in ASD is growing and there are now several clinical and anecdotal reports that the sensory abnormalities that are observed among individuals with ASD involve more than one sensory modality (e.g., Frith, 1989; O'Neill and Jones, 1997; Brock et al., 2002; Happé, 2005). One piece of evidence in support of this notion comes from studies on the multisensory integration (MSI) of speech and emotions as perceived from the face and the voice. Several studies reported that persons with ASD may have less MSI (de Gelder et al., 1991; Bebko et al., 2006; Smith and Bennetto, 2007; Magnée et al., 2008; Mongillo et al., 2008; Russo et al., 2010). As an example, de Gelder et al. (1991) reported that individuals with ASD were normal in auditory speech perception and were unimpaired in silent lipreading, but when the auditory and visual information streams were combined, there was very little effect of the lipread information on auditory speech perception. Magnée et al. (2008) also observed that high-functioning adults with ASD had difficulties integrating heard and lipread speech that could not be attributed to problems in abnormal low-level integration. Possibly, then, people with ASD may have a generalized deficit integrating information from different modalities (Kern et al., 2007; Mongillo et al., 2008; Oberman and Ramachandran, 2008; Foxe and Molholm, 2009; Brandwein et al., 2012).

Others, however, found MSI in people with ASD to be normal (Van der Smagt et al., 2007; Grossman et al., 2009; Foss-Feig et al., 2010). Williams et al. (2004) reported that children with autism normally utilized visual information in identifying auditory speech. Children with autism could also determine—as typically developing (TD) controls did—whether the specific sound of a bouncing ball matched its physical appearance (Mongillo et al., 2008). Equivalent amounts of MSI between an ASD- and TD-group were also reported when participants were asked to detect the direction of two laterally separated beeps in the presence of concurrent visual apparent motion, or the number of flashes when concurrent beeps were present (Keane et al., 2010). When these results are taken together, there is thus no consensus as to what extent MSI is impaired in autism.

Research in ASD's (multi)sensory processing has mainly focused on the integration of higher-level information like speech or faces, but there has been far less interest in the underlying mechanism and the constraints under which information from different modalities is combined. In research on MSI, it is generally agreed that (near) temporal synchrony is *the* most important factor for MSI to occur (e.g., Welch and Warren, 1980; Stein and Meredith, 1993; Radeau, 1994). Intersensory integration thus will only occur if information from the different sensory modalities arrives at approximately the same time in the brain because otherwise two separate events are perceived. However, temporal synchrony between the senses is not straightforward, because there is no evidence of a dedicated sense organ that registers time in an absolute scale. It is well-known that the neural transduction times of the various sensory modalities differ significantly, and the brain thus has to overcome differences in transduction and neural transmission time (Pöppel, 1997; Vroomen and de Gelder, 2004). It is conceivable that if there are fundamental disturbances in the temporal orchestration of multisensory events, this will lead to deficits in multisensory processing as well. Brock et al. (2002) indeed theorized that the critical deficit of MSI in people with ASD may lie in the temporal synchronization among both local and distributed neural networks. These networks can show strong patterns of entrainment in response to a given sensory stimulus (i.e., a focus of activation in one area is soon followed in a strongly time-locked fashion by a focus in a second connected brain area), and this temporal synchronization among brain regions is likely to be critically important in the binding of multisensory stimuli into unified perceptual constructs (Senkowski et al., 2008). A critical question is thus whether people with ASD do indeed suffer from intersensory temporal deficits that may underlie other impairments in MSI.

At present, autism studies on temporal processing are very limited, but some indeed report differences in various aspects of temporal functioning. Szelag et al. (2004) studied temporal processing in the time domain of a few seconds in children with ASD and found important deficits in duration judgments compared to TD children. In a study by Bebko et al. (2006), intermodal perception of audiovisual temporal synchrony in young children with autism was compared with children without impairments and a group of children with other developmental disabilities. The ASD group displayed reduced or no preference at all for synchronous over asynchronous audiovisual speech and non-speech stimuli, possibly because children with ASD are not (yet) sensitive to audiovisual temporal synchrony. Two recent studies, by Kwakye et al. (2011) and Foss-Feig et al. (2010), proposed that individuals with ASD may have an extended window of multisensory temporal binding. The study by Foss-Feig et al. (2010) used the sound-induced double-flash illusion in children with ASD. In this illusion, pairing of a single visual stimulus (i.e., flash) with several auditory stimuli (i.e., beeps) often results in the perception of two or more flashes (Shams et al., 2000). Foss-Feig et al. (2010) reported that children with ASD had this flash-beep illusion over an extended range of stimulus onset asynchronies (SOAs) relative to TD children. Kwakye et al. (2011) also reported an extended window of temporal integration in children with ASD using temporal order judgment (TOJ) tasks with visual, auditory, and audiovisual stimuli. The authors reported no differences in sensitivity for visual temporal order, but thresholds were higher in ASD on the auditory TOJ task. In the multisensory TOJ task, the authors relied on the phenomenon known as temporal ventriloquism (Scheier et al., 1999), where click sounds can improve sensitivity for visual temporal order if the clicks are presented within a certain time window. Children with ASD showed performance improvements over a wider range of temporal intervals than TD children, thus reinforcing the idea that children with ASD have a wider temporal window of MSI.

In this current study we presented adolescents with ASD and TD controls an audiovisual TOJ task to examine their sensitivity of intersensory temporal order in a direct way. Three kinds of stimuli were used that are known to differ on a number of potentially relevant dimensions: a flash/beep, a video of a handclap with the corresponding sound, and a video of a face articulating a syllable with the corresponding speech sound. The asynchrony between the audio and video was varied, and participants judged whether the auditory stimulus came “early” or “late” with respect to the video. By using different stimuli (flash/beep, handclap, speech), we varied the complexity of the stimuli that allowed us to examine whether people with ASD suffer from a general or a more specific impairment in audiovisual temporal processing. Previous studies with TD participants have shown that people are more sensitive to audiovisual timing differences of artificial stimuli than audiovisual speech (Dixon and Spitz, 1980) and that judging temporal order in audiovisual speech is particularly difficult, possibly because it lacks fast visual and auditory transients that can serve as temporal markers (Stekelenburg and Vroomen, 2007). The handclap condition was expected to be relatively easy for TD participants because it not only contains a fast audiovisual transient, but also visual information that predicts sound onset that may serve as a temporal anchor (Vroomen and Keetels, 2010). If individuals with ASD have impairments in action understanding (Smith and Bryson, 1994; Iacoboni and Dapretto, 2006; Zalla et al., 2010) that disrupt their ability to predict others' actions, one may expect individuals with ASD to profit less from this predictive information in the handclap condition. Finally, people with ASD may have specific problems judging audiovisual synchrony in faces because they are especially handicapped in processing socially relevant stimuli (Kanner, 1943; Swettenham et al., 1998; Klin et al., 2002; Bebko et al., 2006; Riby and Hancock, 2008). Numerous studies have indeed demonstrated that individuals with ASD exhibit abnormalities in perceiving and attending facial and social stimuli (Osterling and Fawson, 1994; Dawson et al., 1998; Schultz et al., 2000; Golarai et al., 2006). We therefore expected that people with ASD may have specific deficits judging temporal order of audiovisual speech because it is both a complex and social stimulus.

## Materials and methods

### Participants

Sixteen high-functioning adolescents with ASDs were included, 11 males and 5 females, ranging in age between 16 and 22 years (mean age = 19.2, *SD* = 2.4). The clinical participants were all in residential care and recruited from “De Steiger” (part from mental health institution “Yulius”), a residential unit in Dordrecht, The Netherlands, serving patients with ASD exclusively. Ten adolescents were administered the Wechsler Adult Intelligence Scale (WAIS-III) and six the Wechsler Intelligence Scale for Children (WISC-III) (see Table 1 for individual demographics per group). The severity of autistic symptoms was quantified with a checklist of the 12 DSM-IV (APA, 1994) diagnostic criteria (sub A) for 299.0 autistic disorder (see also Teunisse et al., 2001; Berger et al., 2003). Based on this checklist and on the expertise of a professional clinical team, two of the participants in the ASD group met DSM-IV criteria for autistic disorder, 10 for Pervasive-Developmental-Disorder Not-Otherwise-Specified (PDD-NOS) and four for Asperger's disorder (see also Table 2 for overall group demographics). Participants in the TD-control group were recruited from Tilburg University and were non-psychiatric, eleven males (mean age = 19.6 years) and five females (mean age = 18.4 years), age range 18–22 years. Both groups were matched on age, gender and IQ. None of the participants had a history of serious medical, neurological or psychiatric illness (apart from ASD), seizure disorder, trauma, or use of medication affecting the nervous system. All reported normal or corrected-to-normal vision and hearing and were tested individually. The ASD group received gift vouchers for their participation, the TD group received course credits in return. All participants were naïve to both the experimental procedure and the purpose of the study and gave written consent prior to participating (in case of immature participants, written consent was also given by parents). The study was carried out in accordance with the ethical standards of the Declaration of Helsinki and was approved by the Medical Review Ethics Committee of the St. Elisabeth Hospital, Tilburg, The Netherlands.

**Table 1:** **Individual demographics and Just Noticeable Difference (JND) in ms per group**.

**Sub**	**Age**	**Gender**	**Diagnosis**	**VIQ**	**PIQ**	**TIQ**	**JND Speech**	**JND Handclap**	**JND Flash**
**ASD GROUP**									
X1	19	M	AUTISM	110	85	98	182.14	66.78	85.40
X2	20	F	AUTISM	102	99	100	202.69	70.22	125.38
X3	19	M	PDD-NOS	81	87	83	150.69	173.64	215.35
X4	18	M	PDD-NOS	89	79	83	229.21	74.27	145.96
X5	16	M	ASPERGER	114	112	115	320.7	130.64	106.77
X6	16	M	ASPERGER	107	117	114	102.09	86.54	100.18
X7	18	F	PDD-NOS	121	140	134	90.37	183.55	206.65
X8	20	M	PDD-NOS	109	123	116	65.64	66.94	72.07
X9	20	M	ASPERGER	108	104	107	61.86	51.72	102.54
X10	21	M	ASPERGER	94	104	98	113.59	61.09	63.86
X11	16	F	PDD-NOS	129	109	115	98.01	60.49	85.29
X12	16	F	PDD-NOS	107	102	106	110.87	81.21	127.14
X13	22	M	PDD-NOS	115	96	107	132.28	66.19	89.61
X14	22	M	PDD-NOS	92	99	98	140.17	53.91	71.06
X15	20	M	PDD-NOS	98	99	97	98.87	66.81	76.63
X16	24	F	ASPERGER	132	119	128	79.30	70.61	129.87
**TD GROUP**									
X20	18	M	–	122	113	120	180.62	100.07	103.37
X21	18	F	–	110	108	109	92.49	75.61	65.12
X22	18	F	–	114	109	112	99.53	66.20	55.72
X23	18	M	–	110	104	107	66.10	56.71	56.26
X24	21	M	–	127	101	117	71.51	54.20	63.53
X25	22	M	–	90	107	96	67.50	60.91	115.98
X26	18	F	–	102	100	101	91.17	55.93	85.10
X27	20	M	–	109	113	111	93.32	57.05	104.27
X28	18	F	–	113	101	108	135.55	63.42	89.48
X29	18	M	–	122	111	119	96.56	65.39	65.80
X30	21	M	–	99	94	96	137.97	98.10	90.67
X31	20	M	–	106	108	107	176.26	107.12	77.84
X32	20	F	–	97	93	94	129.75	56.33	147.56
X33	19	M	–	103	90	97	97.65	79.89	72.56
X34	19	M	–	114	85	101	123.63	91.13	122.87
X35	20	M	–	111	110	111	52.36	53.13	60.71

**Table 2:** **Overall demographics and comparison per group and mean Just Noticeable Difference (JND) and Point of Subjective Simultaneity (PSS) in ms and Standard deviations per condition**.

	**ASD group**		**TD group**		**Comparison**	
	**Mean**	**SD**	**Mean**	**SD**	***t***_**(30)**_	***p***
Age (in years)	19.2	(2.4)	19.3	(1.3)	0.09	0.93
Verbal IQ	106.8	(13.9)	109.0	(9.7)	0.53	0.60
Performal IQ	104.6	(15.4)	103.3	(8.9)	−0.31	0.76
Total IQ	106.2	(14.1)	106.6	(8.4)	0.11	0.92
**Medication**	***N***	**Mean daily dosage**				
Clonidine	1	0.125 mg				
Paroxetine	1	20 mg				
Sertraline	2	75 mg				
Risperdal	1	1.5 mg				
Alprazolam	1	0.25 mg				
**Diagnoses**	***N***					
Autism	2					
PDD-NOS	9					
Asperger	5					
**JNDs PER CONDITION IN ms**						
Speech	136.2	(68.5)	107.0	(37.6)		
Handclap	86.0	(41.5)	71.3	(18.3)		
Flash/beep	112.7	(45.1)	86.0	(26.8)		
**PSSs PER CONDITION IN ms**						
Speech	11.7	(92.4)	42.2	(77.3)		
Handclap	24.0	(64.2)	15.9	(47.5)		
Flash/beep	27.0	(90.0)	65.1	(90.7)		

### Stimuli

**Figure 1:**
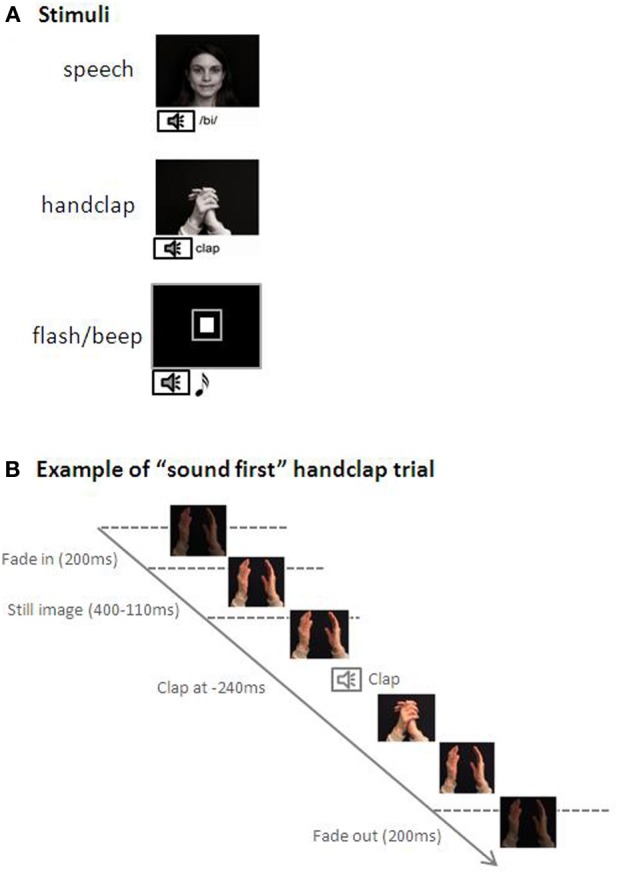
**(A)** Examples of the stimuli used in the three conditions; speech, handclap, and flash/beep. **(B)** Depiction a “sound first” [the sound (handclap) is presented at −240ms] trial in the handclap condition.

Visual stimuli were presented on a 15' laptop monitor [Dell Inspiron 6000, controlled by E-Prime (Psychology Software Tools, Inc.; www.pstnet.com/eprime)] positioned on eye-level, approximately 60 cm in front of the participants. The sounds came from the laptop speakers. There were three types of stimuli: flash/beep, handclap, and speech (see Figure 1A). The flash/beep condition started with the presentation of a gray placeholder (diameter of 3.5 cm) against a dark background in the middle of the screen. After 1000–1500 ms either a 7 ms sound burst of 69 dB(A) or a white square (diameter of 1.5 cm) in the placeholders position were presented with variable SOAs (sound first or flash first). The speech stimulus consisted of the pronunciation of the syllable/bi/by a Dutch female speaker whose face was entirely visible on the monitor. In the handclap condition, a single clap of two hands was presented. The videos were presented at a rate of 25 frames/s. The duration of the videos was 3 s, including a 200 ms fade-in and fade-out, and a still image (400–1100 ms) at the start. The duration of the auditory sample was 325 ms for/bi/and 120 ms for the handclap (for more details on these stimuli see Stekelenburg and Vroomen, 2007, who originally recorded and used these stimuli). The sound pressure level of /bi/ was 63 dB(A) and 67 dB(A) for handclap (see Figure 1B). The SOA between the auditory and visual part of each stimulus stimuli varied in 10 steps (±320, ±240, ±160, ±80, ±40ms, with negative values indicating sound first). This resulted in 10 unique trials each randomly presented 16 times in two blocks of 80 trials each for each of the three stimulus conditions. The three stimulus conditions were blocked and presented in an ABCCBA design, with stimulus order counterbalanced across participants. The SOA varied randomly within each block.

### Procedure

Participants were individually tested in a quiet test room (either at Tilburg University or in “De Steiger” clinic, Yulius). The participants' task was to judge whether the sound came “early” or “late” relative to the visual stimulus. Responses were given by pressing one of two keys (“sound early,” “sound late”) on a response box. Responses were unspeeded with emphasis on accuracy. A practice session preceding the test was given in which trials were presented with the two longest SOAs for each condition. During practice, participants received feedback (“wrong” or “correct”) after each trial. Practice continued until six consecutive correct answers were given. Then testing started without feedback.

## Results

**Figure 2:**
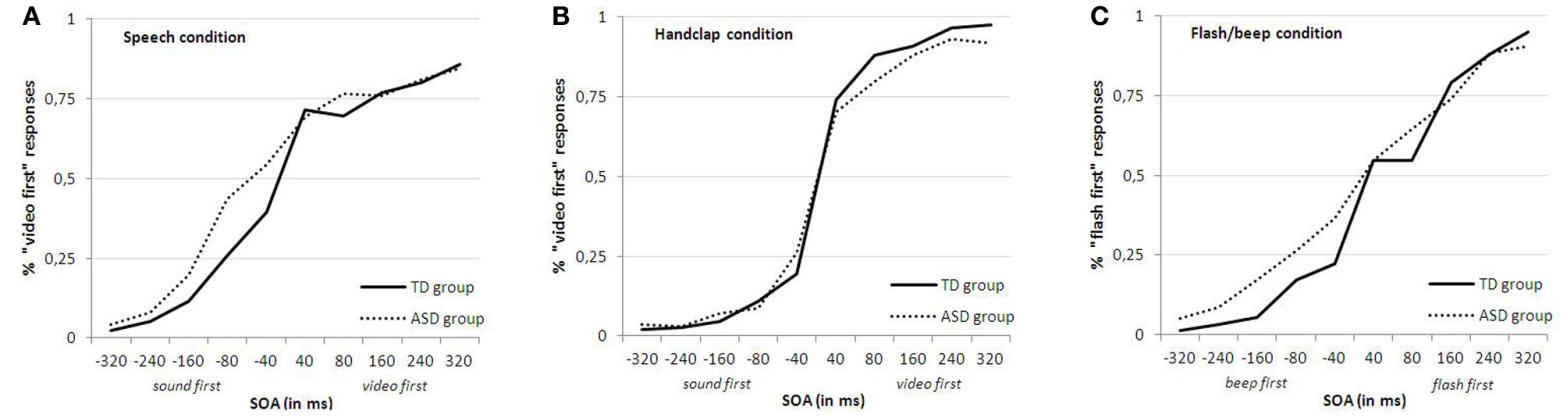
**Averaged raw data of both the TD and ASD group for each condition (“speech **(A)**,” “handclap **(B)**,” and “flash/beep **(C)**”)**.

**Figure 3:**
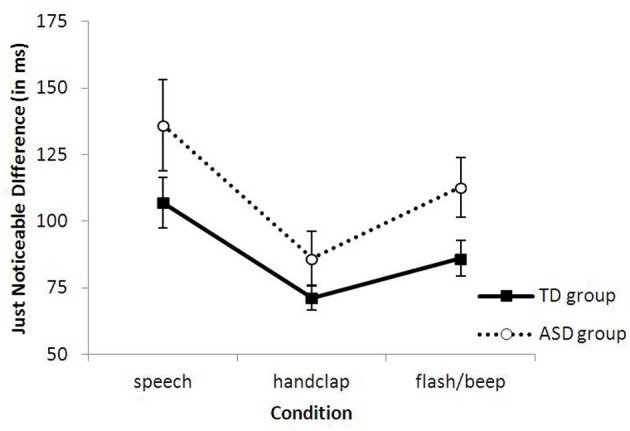
**Group-averaged JNDs as a function of the interval between the sound and video and (bars represent 1 standard error of the mean)**.

Trials of the practice session were excluded from analyses. The individual proportion of *sound-early* responses was calculated for each combination of stimulus condition and SOA, and then converted into equivalent Z-scores (for averaged raw data of both groups for each condition, see Figure 2). For both groups JNDs were initially computed by fitting both logistic and linear functions. The results of the logistic and linear slopes and JNDs were equivalent. The linear function provided a significantly better fit to the data of the three conditions than the logistic function for both groups, and was therefore used as the principle function. The mean and standard deviations of R^2^ for the control group were 0.79 (±0.08), 0.81 (±0.08) and 0.85 (±0.11) for the three conditions (speech, handclap and flash/beep) and 0.76 (±0.14), 0.82 (±0.10) and 0.81 (±0.15) for the ASD group. For each condition, the best fitting straight line was then calculated over the ten SOAs. Two subjects (one from each group) were excluded from further analyses, because their results did not conform to a typical s-shaped function. The lines' slopes and intercepts were used to determine the just noticeable difference (JND = 0.675/slope) and the point of subjective simultaneity (PSS). The JND represents the smallest interval between two stimuli needed by participants to correctly judge which stimulus came first. A smaller JND thus represents good sensitivity as smaller stimulus differences are required for correctly judging temporal order. The PSS represents the average interval by which one stimulus had to lead the other for being perceived as simultaneous. The group-averaged JNDs for each condition are presented in Figure 3. As is clearly visible, the ASD group had overall larger JNDs than the TD group. This indicates that individuals with ASD were less sensitive to judge audiovisual temporal synchronies. This was confirmed in a 2 (group) × 3 (condition) ANOVA on the JNDs. There was a main effect of group *F*_(1, 31)_ = 4.399, *p* < 0.05, η *p*^2^ = 0.13, indicating that, on average, the ASD group had larger JNDs than the TD group (group averages of 116.6 ms and 88.1 ms for ASD and controls, respectively). There was also a main effect of condition *F*_(2, 30)_ = 12.058, *p* < 0.001, η *p*^2^ = 0.29, because sensitivity differed among the three different stimuli, while the theoretically important Group × Condition interaction was not significant, *F* < 1. As also visible in Figure 3, both groups showed the smallest JNDs (best sensitivity) in the handclap condition. Independent *t*-tests across groups comparing the three conditions confirmed that the difference in JND (43 ms) between the speech and handclap condition, *t*_(31)_ = 4.619, *p* < 0.001, and the 20.4 ms difference between the flash/beep and handclap condition, *t*_(31)_ = −4.067, *p* < 0.001, were significant. The difference in JND (22.3 ms) between the speech and flash/beep condition was also significant, *t*_(31)_ = 2.093, *p* < 0.05. To summarize, we succeeded in creating audiovisual stimuli that varied in their difficulty of judging the temporal order of their components. Individuals with ASD were, in general, less sensitive perceiving small audiovisual timing differences than controls, but they were not specifically impaired with audiovisual speech.

For completeness, similar analyses were also run on the PSSs (see Table 2). A 2 (group) × 3 (conditions) overall ANOVA on the PSSs showed that there was no effect of Group (*F* = 1.137, *p* > 0.05), Condition (*F* = 1.17, *p* > 0.05), and no interaction between the two factors (*F* < 1). The point at which the audiovisual stimuli were perceived to be maximally synchronous thus did not differ between group and stimuli.

## Discussion

Here we examined sensitivity of audiovisual temporal asynchronies in adolescents with ASD and TD controls in an audiovisual TOJ task. The results showed that the ASD group had larger JNDs (= lower sensitivity) than the TD group, indicating that the ASD group had more difficulty judging audiovisual synchrony. Furthermore, we hypothesized that people with ASD might be specifically impaired when social stimuli were involved, and therefore used different stimulus classes (audiovisual speech, handclap and flash/beep). However, there was no trace of a specific impairment, as the JNDs of both groups were equally affected by the different stimuli. This thus suggests that people with ASD may suffer from a more general impairment in audiovisual temporal processing.

Our findings fit a study by Bebko et al. (2006) who showed that children with ASD had impairments in the detection of violations of temporal synchrony of audiovisual linguistic stimuli if compared to TD children and children with non-autistic developmental delays. They used a preferential looking paradigm in which the children viewed two screens displaying identical video tracks, but one offset from the other by 3 s, and with the single audio track that matched to only one of the displays. Even though their study showed (very small) contrasting results for non-linguistic stimuli (which can be explained by choice of paradigm and the essential difference in timing durations of the asynchrony in audiovisual stimuli, 3 s compared to our range of 320–340 ms), there are indications of impaired temporal sensitivity for synchrony.

Grossman et al. (2009) wanted to test whether high-functioning ASD adolescents were able to integrate AV information of meaningful, phrase-length language in a task of onset asynchrony detection. They found no significant differences between adolescents with ASD and their TD peers in accuracy of onset asynchrony detection. The authors used video clips of complete phrases, using simple, commonly occurring words. The clips were manipulated to have the video precede the corresponding audio by audio delays from 120–500 ms. Like the Bebko et al. (2006) study, the delays in this study were substantially larger compared to our study (40–320 ms). This temporal component could be an explanation for the contrasting results between the studies. As Figure 2 of our data shows, the ASD group scores near 85-95% correct on the large SOAs (at least ±320 and ±240). This percentage drops at the smaller SOAs (just like in the TD group). These results show that the ASD group is capable of judging temporal asynchrony, but their larger JNDs reveal that they need more time between the audiovisual stimuli to do so (but in a much smaller timeframe than the studies described before). A possible interpretation of these results could be that people with ASD continue to bind two stimuli as part of one event.

An extended window of temporal binding of two intermodal stimuli in people with ASD has also been proposed by Kwakye et al. (2011) and Foss-Feig et al. (2010). Results of both their flash-beep illusion and TOJ studies revealed that children with ASD have altered multisensory temporal function as they showed extended illusion ranges in performance in the multisensory tasks. For example, in the TOJ task performance gains of the children with ASD manifested themselves as improvements in accuracy and as faster responses relative to the unisensory (i.e., visual-only) baseline condition across an increased range of multisensory delays [important to mention here is that the SOAs in these studies are comparable with those in our study (a range of 0–500 ms)]. It seems conceivable that the diminished sensitivity to temporal asynchrony we found here could result in an enlarged multisensory temporal binding window. Alternatively, though, it might also be a result of some temporal binding deficit, as proposed by Brock et al. (2002). They suggest that activities within networks of interconnected sensory areas are not as strongly correlated in ASD, resulting in disruptions in the binding of perceptual information. Kwakye et al. (2011) further speculate that it may be the case that these neural signals are not so drastically uncorrelated as to cause decoupling across regions (as initially hypothesized by Brock et al., 2002), but instead occur in such a way as to necessitate an extended temporal binding window within which two stimuli can continue to be bound as part of one event. Clearly, further research is needed for additional information on these mechanisms and theories on networks connections.

Interestingly, our results show no differences in the judgment of the audiovisual temporal order of specific stimuli between the two groups. Both groups performed best with clapping hands, followed by the artificial beep-flash, and worse with audiovisual speech. These findings concur with the results of Stekelenburg and Vroomen (2007). They used the same TOJ task (except for the artificial condition) and found that JNDs for non-speech events (clapping hands) were smaller than for speech (facial condition). This indicates that the temporal relation between audition and vision of the handclap was more precisely defined. The authors also pointed out that the clapping hands contained more anticipatory visual motion (280 ms) than the speech stimuli (160 ms), and faster transient onsets in audition and vision. They proposed that judging temporal order in audiovisual speech is particularly difficult because it contains less visual anticipatory motion and lacks fast auditory and visual transients. Apparently, predictive information can be used by individuals with ASD in this task, despite their putative impairments in action understanding.

We also hypothesized that people with ASD might have specific problems judging audiovisual temporal order in audiovisual speech because of the social component. Numerous studies reported that individuals with ASD exhibit abnormalities in facial and social stimuli (e.g., Osterling and Fawson, 1994; Dawson et al., 1998; Schultz et al., 2000; Golarai et al., 2006). However, we found no such impairments here with faces and, arguably, the hand clap. An explanation might be that the participants' task in our experiment did not involve speech comprehension, face recognition, facial expression, or emotion-reading. Participants were presented a short non-word “bi” as pronounced by a female face and they only had to judge whether the sound came before or after the lips moved. The spoken stimuli thus had no further meaningful content, and this focus on low-level aspects of the stimulus might overshadow its social relevance. Another explanation might be more temporal, as the duration of our videos was relatively short (3 s) compared to other studies (e.g., Dawson et al., 1998; Bebko et al., 2006). Participants were thus exposed to much shorter fragments of faces which may ease processing load.

There are some obvious limitations in our study. Firstly, we only investigated a very specific group of high-functioning adolescents with ASD and it remains to be examined whether this can be generalized to other subtypes of ASD. Interpreting the results is also complicated by the heterogeneity of the disorder, even within each subtype. Therefore, our results may not apply to other subpopulations of ASD such as children, adults or lower-functioning people with autism. Additional research will have to consider how temporal intersensory processing varies across subpopulations and how individuals within these groups relate to those with typical development who are typically developed or to those who are both developmentally impaired and non-autistic.

Although the exact causes of our findings are speculative, they are in line with the majority of ASD studies on MSI which show that individuals with ASD have altered MSI (de Gelder et al., 1991; Bebko et al., 2006; Kern et al., 2007; Smith and Bennetto, 2007; Magnée et al., 2008; Mongillo et al., 2008; Oberman and Ramachandran, 2008; Foxe and Molholm, 2009; Russo et al., 2010), or altered multisensory temporal function (Foss-Feig et al., 2010; Kwakye et al., 2011). Further research is clearly needed to examine and characterize multisensory processes in ASD in more detail and this may ultimately lead to a broader and better understanding and diagnosis of this disorder.

### Conflict of interest statement

The authors declare that the research was conducted in the absence of any commercial or financial relationships that could be construed as a potential conflict of interest.
